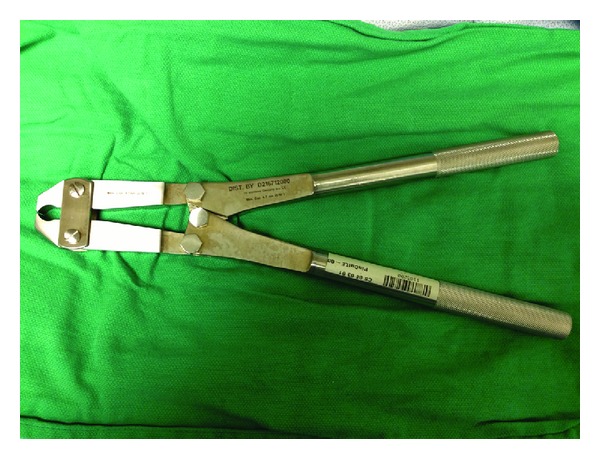# Removal of a Penile Constriction Device with a Large Orthopedic Pin Cutter

**DOI:** 10.1155/2014/347285

**Published:** 2014-02-20

**Authors:** Avinash Chennamsetty, David Wenzler, Melissa Fischer

**Affiliations:** ^1^Department of Urology, Beaumont Health System, Royal Oak, MI 48073, USA; ^2^Department of Urology, Beaumont Health Systems and Oakland University William Beaumont School of Medicine, Royal Oak, MI 48073, USA

## Abstract

Penile strangulation is an infrequent clinical condition that has widely been reported. It usually results following placement of a constriction device to enhance sexual stimulation. Early treatment is essential to avoid potential complications, including ischemic necrosis and autoamputation. We describe the use of a Large Orthopedic Pin Cutter to remove a metal penile constriction device in the Emergency Department (ED). This case report describes the relatively safe technique of using an instrument available in many hospitals that can be added to the physician's arsenal in the removal of metal constriction devices.

## 1. Introduction

Penile strangulation is a rare and challenging clinical presentation that usually requires urgent management. It generally follows self-placement of a constriction device for enhancing sexual stimulation or by persons suffering from psychiatric illness [1]. The condition has been widely reported in the literature with the first reported case in 1755 [2]. The choice of method for removal depends on the type and size of the metal object, incarceration time, trauma grade, and availability of equipment [3]. Early treatment is essential to avoid potential complications including ischemic necrosis, diminished sexual function, and even amputation [4, 5].

Removal of these objects often produces considerable anxiety in the patient as well as the physician and presents a great challenge to the latter. Ideally, one should employ a method that is noninvasive and quick. There have been various reports of removal of these devices using various saws, grinders, and other motorized tools, sometimes requiring assistance from professionals such as firemen, facility engineers, and jewelers [6–12]. We describe the use of a large orthopedic pin cutter, an instrument available in many hospitals, for the removal of a metal constriction device.

## 2. Case Report

**Figure 1:**
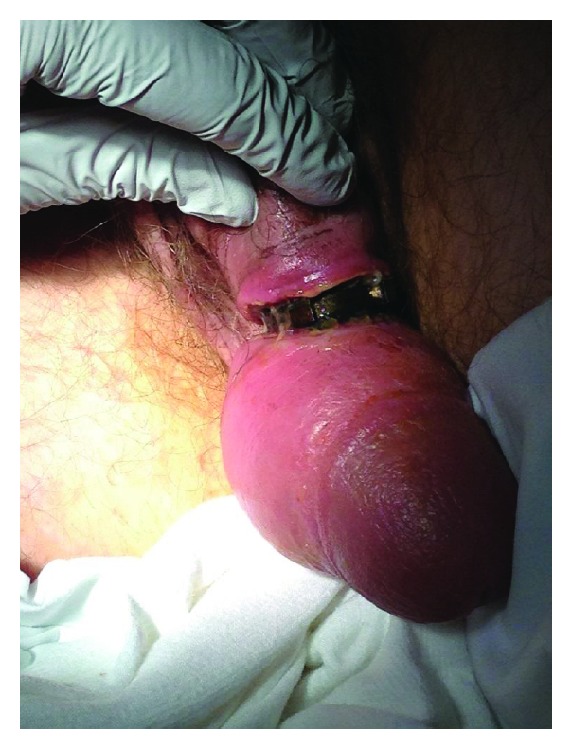


A 49-year-old man presented to the ED complaining of pain and swelling of his penis secondary to an incarcerated penile constriction device placed 9 days before presentation. The device was placed by the patient himself for autoerotic motive. He later found that he was unable to remove it. He had no history of psychiatric illness and did not seek any medical attention prior to the ED visit. He complained of increasing swelling and severe pain. He was able to urinate but had a decreased force of stream. Physical exam revealed a tightly encircling metallic ring with peripheral cogs placed on the midshaft of the penis causing severe penile engorgement and edema (Figure 1). The metal appeared to be a very hard alloy with thickness measuring 5–7 mm depending on the location. The penile skin under the ring was excoriated and necrotic. Due to the incarceration time, degree of necrosis, and significant distal edema, simple lubrication, compression, and manual removal were not an option for fear of amputation. Manual and electric ring cutters were used, but after several attempts, we were unable to do more than scratch the surface of the metal ring.

**Figure 2:**
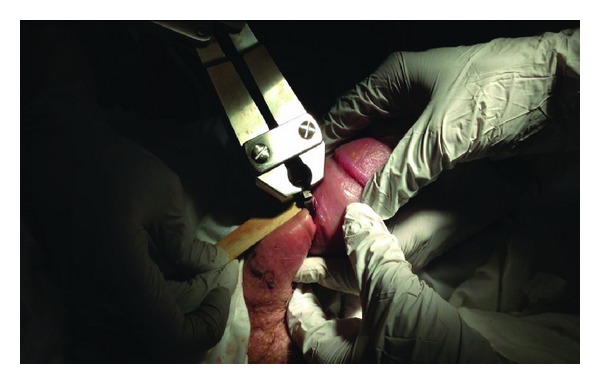


**Figure 3:**
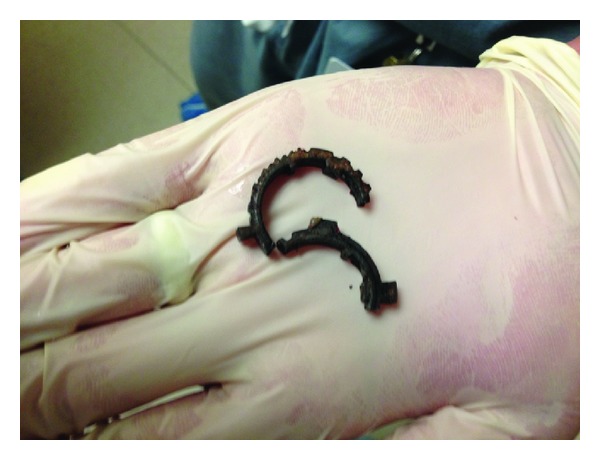


At this point, we contacted the orthopedic central parts depot and obtained a Large Orthopedic Pin Cutter. The patient was given procedural sedation and a tongue depressor was placed beneath the metal ring to provide soft tissue protection (Figure 2). Using the pin cutter, enough force was generated in one attempt to snap the ring into two separate pieces (Figure 3). The penis was cleansed with saline and a nonadherent antimicrobial was applied. The patient was then catheterized with a 16 Fr Foley catheter with clear urine return and was admitted for observation and care. After 24 hours, penile edema slightly improved but still persisted. The urethral catheter was removed after two days, following which, the patient voided satisfactorily. He was discharged with advice to maintain local hygiene and apply a topical antibiotic over the area. On two-week followup, penile edema had subsided completely and the overlying soft tissue was healing well by secondary intention. He had normal voiding and noticed return of nocturnal erections. The patient failed to follow up for subsequent visits.

## 3. Discussion

The complications of penile strangulation depend on several factors including the degree of constriction and the time elapsed until presentation. Several authors have attempted to grade such injuries. Bhat et al. developed a five-tier grading system [13]. Grade I caused only edema of distal penis without skin ulceration, while Grade II occurred with injury to the penile skin, distal penile edema, and the presence of penile paresthesia. Grade III involved injury to the skin and urethra but no urethral fistula. When a fistula did occur, this is classified as Grade IV. Gangrene, necrosis, or complete amputation is considered a Grade V injury. Grade I and II injuries are not associated with urethral trauma and can be managed by temporary urethral catheterization, as was done in this case, allowing the penile edema and pain to subside. Another grading system by Silberstein et al. categorized injuries into low- and high-grade [3]. Low-grade injuries are likely to require no further surgical intervention after the constriction device has been removed, while high-grade injuries do require it. The current case would be classified as a Bhat grade II injury or a Silberstein low-grade injury.

Removal of a penile constriction device by cutting is the most common method described in the literature, but various more invasive techniques have been described such as string technique and its modification; penile aspiration techniques; and surgical excision of the penile skin and Buck's fascia [7, 14, 15]. In the case described above, a Large Orthopedic Pin Cutter (Figure 4), which measured approximately 21′′ in length allowing for powerful leverage and had a maximum capacity of 4.7 mm (3/16′′), was used. Even though the thickness of the metallic ring exceeded the maximum capacity of the instrument, which can be as high as 6.35 mm (1/4′′) depending on the manufacturer, the ring was cut with ease. However, these instruments may play a limited role in the cutting of bulky metabolic objects (pipes, metallic ball bearings, etc.). Nonetheless, most hospitals equipped to handle orthopedic injuries or trauma should have these or a similar instrument available.

We were able to conduct this procedure in the ED with the patient under sedation. Extreme care should be taken to avoid any iatrogenic injury to the genitalia. The use of the wooden tongue depressor proved to be an adequate barrier to protect the soft tissue. However, many other devices have been described in the literature, including metal tongue blades, plastic guards, and even laryngoscope blades [7, 10, 16, 17]. These should be used if available as they offer more of a barrier and provide more protection than the wooden tongue blade we used.

## 4. Conclusion 

Penile strangulation is a serious injury necessitating urgent attention and timely removal. It proposes a difficult challenge to the physician where surgical resourcefulness is necessary to have a successful outcome. We propose the use of orthopedic pin cutters as a valuable tool in the physician's armamentarium in the removal of these devices.

## Figures and Tables

**Figure 4:**